# Editorial: New mechanistic insights into cancer precision medicine

**DOI:** 10.3389/fphar.2023.1159703

**Published:** 2023-02-16

**Authors:** Hang Fai Kwok

**Affiliations:** ^1^ Cancer Centre, Faculty of Health Sciences, University of Macau, Taipa, Macau SAR, China; ^2^ Department of Biomedical Sciences, Faculty of Health Sciences, University of Macau, Taipa, Macau SAR, China; ^3^ MoE Frontiers Science Center for Precision Oncology, University of Macau, Avenida de Universidade, Taipa, Macau SAR, China

**Keywords:** immunotherapy, targeted therapy, therapeutic antibody, chemotherapy, novel bioactive molecule, biomarker, drug resistance

Anticancer drugs’ specificity and precise mechanism are crucial in reducing the severity of side effects associated with the drugs’ use. However, due to the similarity between cancer cells and non-cancerous (normal) cells in our body, anticancer agents are generally toxic to normal cells that can cause numerous side effects, some of which are life-threatening. Those adverse effects may require that the drug dosages are reduced, or the drug regimen be changed to make the drugs tolerable to the patients. In the last decade, many studies have been performed to understand cancer’s response by considering the individual aberrant genetic background, which yields essential information for precision medicine. Furthermore, exploring the coping mechanisms of the malignancy after exposure to drug treatments provides hints about compensatory strategies resulting in drug resistance and how to tackle the “Achilles heel” of cancer progression with a recently developed idea on a precision medicine approach.

In this Research Topic, we brought together leading researchers to exchange and share their findings on the hot topics of the latest mechanistic insights into cancer precision medicine. There are three review articles in this Research Topic. One review is a Long Non-coding RNA study on the cancer radioresistance/radiosensitivity (Wu et al.). The authors performed a systematic review and summarised that lncRNAs are important regulators in tumor radioresistance/sensitivity. Different lncRNAs may participate in the radioresistance with the same regulatory paradigm. In addition, the same lncRNAs may also participate in the radioresistance differently. Future research should focus more on comprehensively characterising the mechanisms of lncRNAs in tumor radioresistance to help us identify corresponding novel biomarkers.

The second review article systematically analysed the recent progress of antibody–drug conjugates (ADCs) in advanced gastric cancer therapy (Wand et al.). Based on their analyses, ADCs are valid and well-tolerated anticancer drugs whose development is a tremendous breakthrough in tumor therapy and the linchpin of AGC treatment. The authors suggest that optimising each ADC component is one of the essential steps for current research in this field. A better understanding of potential modifications can make ADCs individualised and accurate. It is highly believed that the research and development of ADCs will further improve the prognosis of AGC patients.

The third review article dissected the current landscape of personalised clinical treatments for triple-negative breast cancer (TNBC) (Zhang et al.). Since tumors have a massive number of molecular alterations, it may be arduous to achieve clinical efficacy by targeting just one or several of the mutation. Nevertheless, there is no denying that precision oncology brings hope to treating TNBC. Based on TNBC molecular classification, it is necessary to mine therapeutic targets within each subtype and formulate corresponding therapeutic strategies. Future research still needs to develop highly effective targeted drugs and identify relevant biomarkers to evaluate therapeutic benefits. This review highlighted the latest knowledge about these potential TNBC medicinal drugs, which will provide comprehensive insights into the personalised therapeutic strategies and options for combating TNBC.

In addition, Ge et al. studied EPM2A as a protective factor in prostate cancer with evidence from a real-world patient cohort. Their study identified the prognostic/predictive value of EPM2A in prostate cancer *via* a bioinformatics method. Furthermore, patients with higher EPM2A are more sensitive to immunotherapy. In contrast, patients with lower EPM2A are more suitable for bicalutamide, cisplatin and paclitaxel therapy. In conclusion, Ge et al. constructed a nomogram risk model and wished to offer individual clinical endpoint predictions and optimise personalised treatment for each patient.

In conclusion, the published research and review articles on this Research Topic expand our knowledge of the latest studies in novel prognostic, therapeutic and mechanistic insights into cancer precision medicine and therapy. These latest updated studies and analyses fill the gap hindering the potential use of drug candidates for making a better precision cancer diagnosis, planning treatment and prognosis.

